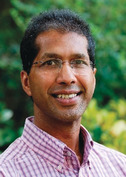# Stereospecific 1,2‐Migrations of Boronate Complexes Induced by Electrophiles

**DOI:** 10.1002/anie.202008096

**Published:** 2020-07-22

**Authors:** Hui Wang, Changcheng Jing, Adam Noble, Varinder Kumar Aggarwal

**Affiliations:** ^1^ School of Chemistry University of Bristol Cantock's Close Bristol BS8 1TS UK

**Keywords:** 1,2-migration, boronate complex, cross-coupling, electrophiles, stereospecific

## Abstract

The stereospecific 1,2‐migration of boronate complexes is one of the most representative reactions in boron chemistry. This process has been used extensively to develop powerful methods for asymmetric synthesis, with applications spanning from pharmaceuticals to natural products. Typically, 1,2‐migration of boronate complexes is driven by displacement of an α‐leaving group, oxidation of an α‐boryl radical, or electrophilic activation of an alkenyl boronate complex. The aim of this article is to summarize the recent advances in the rapidly expanding field of electrophile‐induced stereospecific 1,2‐migration of groups from boron to sp^2^ and sp^3^ carbon centers. It will be shown that three different conceptual approaches can be utilized to enable the 1,2‐migration of boronate complexes: stereospecific Zweifel‐type reactions, catalytic conjunctive coupling reactions, and transition metal‐free sp^2^–sp^3^ couplings. A discussion of the reaction scope, mechanistic insights, and synthetic applications of the work described is also presented.

## Graphical abstract

**Electrophile‐induced** stereospecific 1,2‐migration of boronate complexes has been successfully applied in many asymmetric reactions. Typically, the electrophilic species provides the activation required for the boronate complex to undergo the desired migration of a group from boron to the adjacent sp^2^‐ or sp^3^‐hybridized carbon. Such activating species range from stoichiometric classical electrophiles to transition metal complexes present in catalytic quantities.

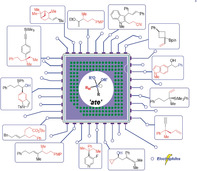

## 1 Introduction

**Scheme 1:**
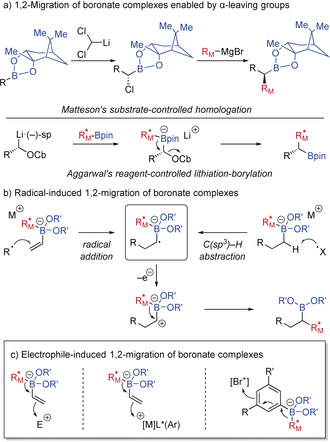
Strategies for stereospecific 1,2‐migrations of boronate complexes. Cb=*N*,*N*‐diisopropylcarbamoyl. R_M_=Migrating group.

Chiral boronic acids and related derivatives are valuable building blocks in modern synthesis as they can be easily prepared with high levels of enantioselectivity.^1^ Crucial to the synthetic utility of organoboron compounds is their ability to be transformed stereospecifically into a range of functional groups.^2^ In general terms, these transformations are initiated by the addition of a nucleophile to the boron atom, resulting in boronate complex formation, followed by a stereospecific 1,2‐migration of a metal migrating group to the adjacent carbon centre.^3^ An example of such a process is the homologation of boronic esters with carbenoids (Scheme 1 a), which has seen wide application in asymmetric synthesis. In this context, Matteson's substrate‐controlled homologation^4^ and Aggarwal's reagent‐controlled lithiation‐borylation^5^ methodologies are particularly noteworthy. Recently, the fields of radical chemistry with stereospecific 1,2‐migration has been shown that radicals next to boronates can be generated by the addition of carbon‐centred radicals to alkenyl boronates^6^ or by α‐C(sp^3^)−H abstraction.^7^ These α‐boryl radical anions can then undergo single‐electron oxidation followed by 1,2‐migration to afford the desired products. This active field has been recently reviewed so will not be discussed further here.^8^

Stereospecific 1,2‐migrations of alkenyl or aryl boronates can be induced by reactions with suitable electrophiles (Scheme 1 c). Although significant and substantial work in this field has been reported, systematic review articles are rare.^9^ Therefore, the aim of this Minireview is to provide an overview of recent developments in electrophile‐induced stereospecific 1,2‐migration of boronate complexes, including Zweifel‐type reactions, conjunctive cross‐couplings, and transition metal‐free sp^2^–sp^3^ couplings. The scope of this review also extends to boronate complexes containing strained σ‐bonds, which exhibit similar reactivity to π‐bonds.

## 2 Stereospecific 1,2‐Migration of Alkenyl Boronates Induced by Electrophiles

### 2.1 Zweifel‐type Coupling Reactions

**Scheme 2:**
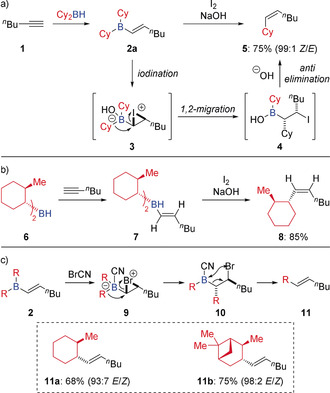
Zweifel olefination: selective synthesis of olefins.

In 1967, Zweifel first reported the stereoselective synthesis of alkenes using organoboron intermediates (Scheme 2 a).^10^ The reaction was initiated by hydroboration of alkyne **1** with dicyclohexylborane, resulting in the formation of alkenyl borane **2 a**, which was then reacted with iodine in the presence of sodium hydroxide, leading to *Z*‐alkene **5**. The reaction proceeds via cyclic iodonium ion intermediate **3**, followed by a stereospecific 1,2‐migration affording β‐iodoborinic acid **4**. This species then undergoes *anti* elimination in the presence of base, which results in an overall inversion of alkene geometry from **2** to **5**. Furthermore, it was later proved that the migrating moiety underwent 1,2‐migration with complete retention of configuration by employing diastereomerically pure borane **6** as a substrate in the reaction (Scheme 2 b).^11^ Considering the stereochemical features of this process, a *syn* elimination (giving the *E*‐alkene) should be possible if the interaction between the β‐halogen and boron of the β‐haloboron intermediate could be enhanced. Indeed, Zweifel demonstrated that *syn* elimination was favoured if a strong electron‐withdrawing group (CN) was introduced on boron, which allowed coordination of the bromide to boron in intermediate **10** and resulted in the formation of *E*‐olefins **11** (Scheme 2 c).^12^

**Scheme 3:**
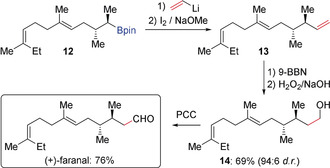
Zweifel olefination in the total synthesis of (+)‐faranal.

**Scheme 4:**
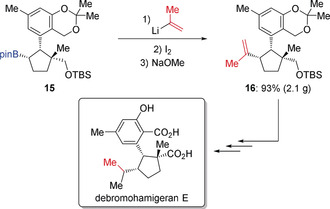
Zweifel olefination in the total synthesis of debromohamigeran E.

The vinyl group is an important functional group, commonly found in natural products and functional materials.^13^ In this context, the Zweifel olefination provides an excellent method to convert a boronic ester into a vinyl group by employing vinyl lithium or the corresponding Grignard reagent.^14^ In 2009, Aggarwal applied this concept to the total synthesis of (+)‐faranal (Scheme 3).^15^ Enantioenriched boronic ester **12** was reacted with vinyl lithium and then treated with iodine and sodium methoxide, which provided alkene intermediate **13**. Without isolation of **13**, in situ hydroboration and oxidation gave alcohol **14** in 69 % yield and with excellent diasteroselectivity. Finally, (+)‐faranal was obtained by oxidation of **14** with pyridinium chlorochromate (PCC). Additionally, this strategy was also successfully used by Morken to introduce an isoprenyl group in the total synthesis of debromohamigeran E (Scheme 4).^16^ Alkene **16** was formed in high yield on a gram‐scale by Zweifel olefination of boronic ester **15** with isopropenyllithium.

**Scheme 5:**
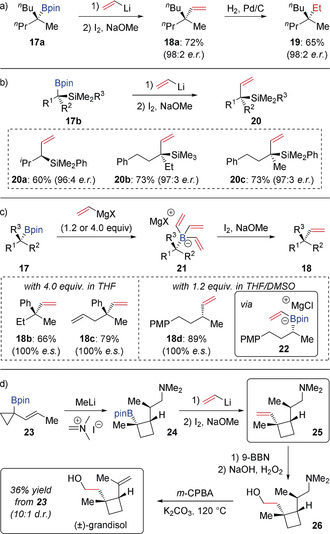
Enantiospecific Zweifel olefinations of secondary and tertiary boronic esters with vinyl lithium or vinyl Grignard reagents. PMP=4‐methoxyphenyl.

Enantioenriched tertiary boronic esters **17** have also been subjected to the same Zweifel olefination conditions to form vinyl‐substituted quaternary stereogenic centers **18** with complete enantiospecificity (Scheme 5 a).^17^ It is noteworthy that allylsilanes **20** could also be obtained in high enantiomeric excess using this protocol (Scheme 5 b).^18^ However, the preparation of vinyllithium typically relies on in situ lithium–tin exchange of tetravinyltin, or lithium‐bromide exchange of vinyl bromide, which reduces its practicality. Therefore, vinyl Grignard reagents, which are easier to handle and commercially available, have also been explored in the Zweifel olefination (Scheme 5 c).^19^ A consequence of changing from vinyllithium to vinyl Grignard reagents, is that magnesium pinacolate is readily formed from Mg^II^ salts and the pinacol ligand on boron. Therefore, upon reaction of a boronic ester with a vinyl Grignard reagent, trivinyl boronate species **21** is formed instead of the mono‐vinyl pinacolato boronate. Normally, this necessitates the use of an excess of vinyl Grignard (4.0 equivalents) but by screening various reaction conditions, it was found that using a mixed solvent system (1:1 THF/DMSO) allowed boronic esters to undergo vinylation using 1.2 equivalents of vinyl Grignard.^20^ Whilst this method shows synthetic utility, it is not suitable for sterically hindered tertiary boronic esters, which makes the higher reactivity of vinyllithium more attractive. This is illustrated in a five‐step synthesis of (±)‐grandisol, where a Zweifel olefination was used to convert tertiary boronic ester **24** into terminal alkene **25** (Scheme 5 d). Subsequent hydroboration/oxidation and Cope elimination provided the natural product in good yield and high diastereoselectivity.^21^

**Scheme 6:**
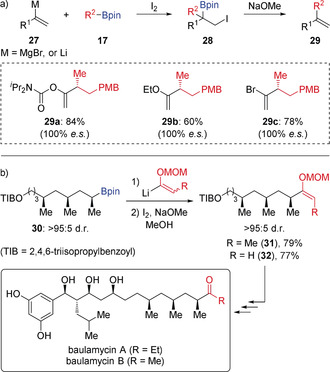
Synthesis of α‐heteroatom‐substituted alkenes by Zweifel olefination and application to the synthesis of baulamycins A and B. PMB=4‐methoxybenzyl.

In the past decade, the scope of the Zweifel olefination reaction has been greatly expanded. For example, α‐heteroatom‐substituted alkenyl metals **27** have been successfully coupled with secondary boronic esters (Scheme 6 a),^20^ which provides great opportunities for application in synthesis as the vinyl ether products **29** can be easily converted into ketones by hydrolysis under mild conditions.^19^ This methodology was used to convert boronic ester **30** into enol ethers **31** and **32** in the synthesis of the reported and revised structures of baulamycins A and B, respectively (Scheme 6 b).^22^

**Scheme 7:**
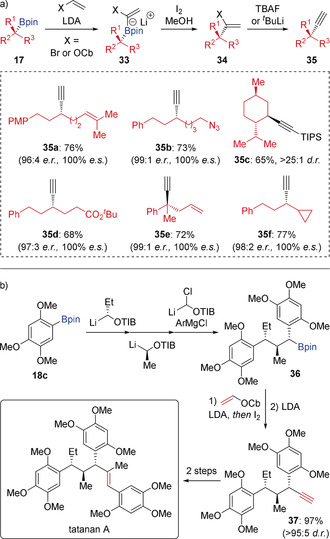
Enantiospecific alkynylation via Zweifel olefination.

The enantiospecific alkynylation of secondary and tertiary boronic esters is an extension to the established Zweifel olefination. In situ α‐lithiation of vinyl bromides or carbamates in the presence of the boronic ester provided boronate complexes **33** that underwent iodine‐induced olefination to give alkenyl bromides or carbamates **34** (Scheme 7 a).^23^ Subsequent base‐induced 1,2‐elimination afforded the alkynylated products **35**. In this reaction, various terminal and silyl‐protected alkynes can be obtained with high enantiospecificity, and a broad range of functional groups (alkenes, azide, alkyne, and ester groups) are tolerated. Furthermore, it was used in the total synthesis of tatanan A, where complex boronic ester **36**—constructed using iterative reagent‐controlled homologation—was employed in an enantiospecific Zweifel‐type alkynylation to afford alkyne **37** (Scheme 7 b).^24^ It should be noted that alkynyl anions cannot be used directly in Zweifel‐type alkynylation since they react reversibly with boronic esters. However, they can used in reactions with boranes or borinic esters.^25^

**Scheme 8:**
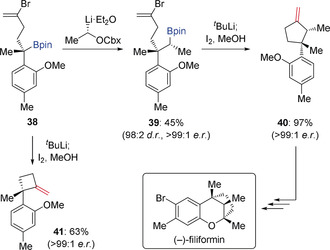
Intramolecular Zweifel olefination with α‐substituted alkenes.

Intramolecular Zweifel olefination has also been achieved, which provides access to methylene cycloalkanes (Scheme 8).^26^ Alkenyl bromide‐containing boronic ester **39**—obtained with high stereoselectivity from **38** by lithiation‐borylation—was treated with ^*t*^BuLi to form an alkenyl lithium intermediate through lithium‐halogen exchange. This species cyclised to give an intermediate cyclic boronate complex. Subsequent treatment with iodine and methanol under Zweifel olefination conditions afforded ring contracted methylene cyclopentane **40** in 97 % yield with 100 % enantiospecificity, which was then transformed into the natural product (−)‐filiformin. This ring contraction methodology was extended to the more challenging synthesis of highly strained methylene cyclobutane **41**, which was obtained in 63 % yield and >99:1 *e.r*.

Since its introduction over 50 years ago, the Zweifel olefination has become a powerful method to transform boronic esters into structurally diverse alkenes with excellent control of alkene geometry. Importantly, by proceeding through a stereospecific 1,2‐migration mechanism, the chiral information of the boronic ester substrate is fully translated to the alkene product. This high level of stereocontrol is often unachievable with metal‐catalyzed Suzuki–Miyaura cross‐couplings, which has resulted in the Zweifel olefination being commonly employed in the synthesis of complex natural products and pharmaceutical intermediates.

### 2.2 Sulfur and Selenium‐Based Electrophiles

**Scheme 9:**
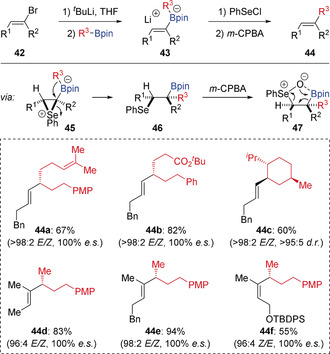
Selenium‐mediated Zweifel‐type olefination via *syn* elimination.

In 2017, Aggarwal reported a modified Zweifel‐type olefination proceeding through a novel *syn* elimination process (Scheme 9).^27^ This was achieved by employing PhSeCl as the electrophile for the selenation of alkenyl boronates **43**, which led to β‐selenoboronic esters **46** through the stereospecific 1,2‐migration ring‐opening of seleniranium intermediates **45**. It was found that *m*‐CPBA was able to chemoselectively oxidise the selenide to give selenoxide intermediate **47**, which underwent *syn* elimination to provide alkenes **44** in high stereoselectivity. DFT calculations showed that the oxygen atom of selenoxide **47** interacts strongly with the boron atom, therefore resulting in a *syn* elimination pathway. This selenium‐mediated olefination showed broad substrate scope in terms of both the boronic esters and the alkenyl lithium reagents (di‐ and trisubstituted), leading to synthetically useful alkene products **44** with high selectivity for retention of olefin geometry.

**Scheme 10:**
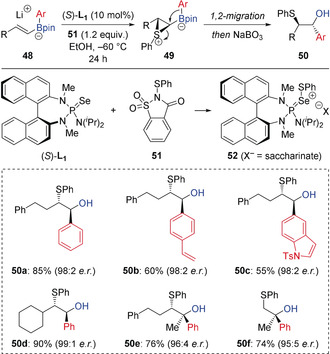
Lewis base‐catalyzed enantioselective electrophile‐induced 1,2‐migration of alkenyl boronates.

In 2018, Denmark reported an alternative chalcogenation‐induced 1,2‐migration of alkenyl boronates (Scheme 10).^28^ Through the use of a chiral Lewis base catalyst in combination with *N*‐(phenylthio)saccharin (**51**) as a source of electrophilic sulfur, an enantioselective sulfenylation was achieved. This provided access to a broad array of enantioenriched *anti* β‐sulfenoboronic esters **50** with two contiguous stereogenic centers with complete diastereoselectivity. Chiral sulfenylating reagent **52**, formed from the nucleophilic addition of chiral selenophosphoramide catalyst (*S*)‐**L_1_** to **51**, is a cationic donor‐acceptor species with a highly electrophilic sulfur atom. Reaction of **52** with alkenyl boronate **48** generates the enantioenriched thiiranium ion **49**, which undergoes 1,2‐migration to generate *anti*‐products **50** with high enantioselectivity.

### 2.3 Transition Metal‐Catalyzed Conjunctive Cross‐Couplings

**Scheme 11:**
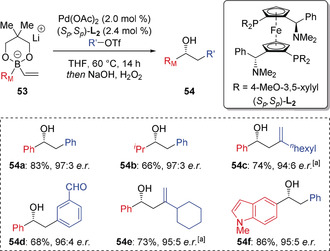
Enantioselective conjunctive cross‐coupling enabled by palladium‐induced 1,2‐migration. [a] Using the pinacol‐derived boronate complex. R_M_=Migrating group.

**Scheme 12:**
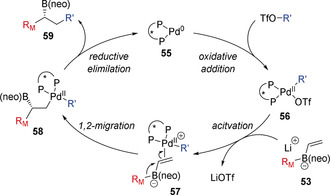
Proposed catalytic cycle of the conjunctive cross‐coupling. neo=neopentyl glycolato. R_M_=Migrating group.

It is known that π‐acidic late transition metal complexes in high oxidation states, such as Pd^II^ and Ni^II^, are highly electrophilic and able to strongly coordinate to π‐bonds. In 2015, Morken reported that such species could interact with the electron‐rich π‐bond of alkenyl boronate complexes, triggering a 1,2‐migration of an alkyl or aryl group on boron (Scheme 11).^29^ Key to the success of this reaction was the use of aryl triflates rather than aryl halides, which generated a more reactive cationic Pd^II^ intermediate, and the use of the Mandyphos ligand **L_2_** to reduce the propensity for β‐hydride elimination of intermediate alkylpalladium(II) intermediates. Furthermore, using a chiral phosphine ligand gave the conjunctive coupling products in good yield and high enantioselectivity. The choice of diol ligand on boron played an important role in determining the enantioselectivity. Interestingly, the optimum diol ligand was found to be dependent on the triflate electrophile, with neopentyl glycol ligands proving optimal for aryl triflates, whereas pinacol ligands provided significantly improved selectivity in reactions of alkenyl triflates. Mechanistically, it was postulated that oxidative addition of Pd^0^ species **55** to an aryl/alkenyl triflate generates the electrophilic Pd^II^ intermediate **56** (Scheme 12). This complexes with alkenyl boronate **53** to form complex **57**, which triggers 1,2‐migration to generate alkyl palladium(II) intermediate **58**. This is followed by reductive elimination, giving the boronic ester **59** and regenerating the Pd^0^ catalyst **55**. The large bite‐angle of ligand **L_2_** limited the undesired β‐hydride elimination of **58**.

**Scheme 13:**
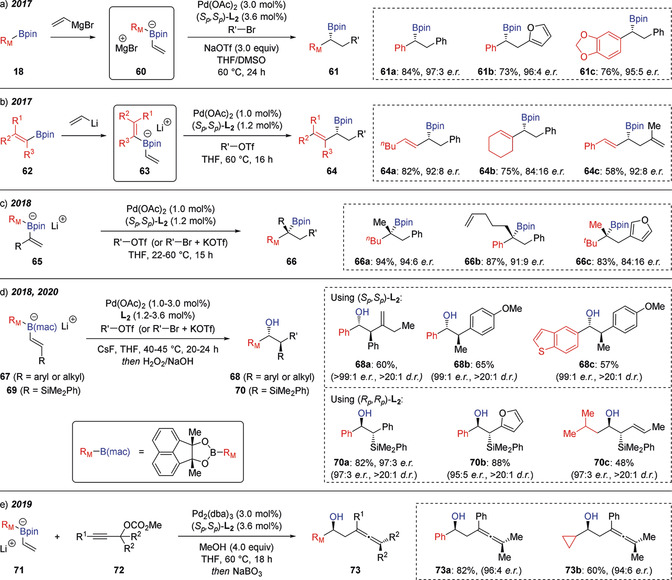
Catalytic conjunctive cross‐coupling reactions enabled by palladium‐induced 1,2‐migration. R_M_=Migrating group.

Morken has built on this discovery with a number of important developments (Scheme 13). Firstly, the reaction has been extended to Grignard reagents instead of organolithiums and to halide electrophiles in place of triflates (Scheme 13 a).^30^ It was found that the conjunctive cross‐coupling was inhibited by halide ions, which had previously limited the use of aryl halide electrophiles. However, this limitation was overcome by using a combination of NaOTf and DMSO as additives, which allowed the formation of cross‐coupled products **61** with high yields and enantioselectivities. The effect of these additives was two‐fold: (i) the NaOTf resulted in precipitation of the sodium halide salt, thus avoiding the detrimental coordination of halide ions to palladium and creating the more electrophilic Pd^II^ complex; and (ii) the combination of NaOTf and DMSO greatly increased the stability of the alkenyl boronate complexes **60** generated from the vinyl Grignard reagent. Conjunctive cross‐couplings between alkenyl boronic esters **62**, vinyllithium, and aryl/alkenyl triflate were next explored (Scheme 13 b).^31^ These reactions proceed through bis‐alkenyl‐boronate complexes **63**, with the Pd^II^ intermediate showing a preference for reaction with the less substituted alkene, and allow access to chiral allylboronic esters **64**. Extension of this approach to boronate complexes derived from α‐substituted alkenyl boronic esters **65** allowed access to highly desirable tertiary boronic esters **66** with good enantioselectivity (Scheme 13 c).^32^ β‐Substituted alkenyl boronic esters **67** were also successfully employed, but required alterations to the boron ligand design to prevent undesired Suzuki‐Miyura‐type reactivity, which was found to dominate with pinacol and neopentyl glycol boronic ester substrates (Scheme 13 d).^33^ A more sterically demanding boronic substituent (mac), derived from acenaphthoquinone, was required to minimize Suzuki–Miyaura coupling and direct the approach of the palladium(II) complex to the more congested β‐carbon, thus enabling access to the conjunctive cross‐coupling products **68** with excellent stereoselectivities. Furthermore, this approach was applied to β‐silyl alkenyl boronate complexes **69** for the efficient construction of *anti*‐1,2‐borosilanes (Scheme 13 d).^34^ Finally, using propargylic carbonates **72** in place of aryl triflates furnished fully substituted β‐boryl allenes with high enantioselectivity (Scheme 13 e).^35^ It was found that a methanol additive resulted in formation of a dimethoxyboronate intermediate through boron ligand exchange, which significantly enhanced both the yield and enantioselectivity of the reaction.

**Scheme 14:**
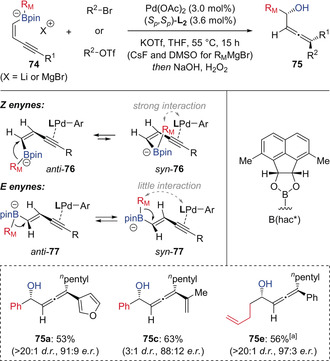
Palladium‐catalyzed enantioselective conjunctive cross‐coupling reactions of enyne boronate complexes. [a] Using B(hac*) instead of Bpin. R_M_=Migrating group.

Morken has since extended this conjunctive cross‐coupling to include enyne‐derived boronate complexes **74**, which give α‐hydroxy allenes **75** after oxidative work‐up (Scheme 14).^36^ Interestingly, enyne boronates derived from *Z*‐alkenes provided α‐boryl allenes with high diastereoselectivity, whereas *E*‐alkene substrates gave low diastereoselectivity. This was rationalized based on the steric interactions between the migrating group and the palladium complex: in the case of the *Z*‐alkene, complex *syn*‐**76** has these moieties in close proximity so they orientate to minimize steric interactions, making *anti*‐**76** the reactive conformer; whereas in the *E* substrate, there is little interaction between the migrating group and the palladium complex in either conformers *anti*‐**77** or *syn*‐**77**, resulting in poor diastereocontrol. For reactions with alkyl migrating groups, substitution of the pinacol ligand on boron for an acenaphthoquinone‐derived boronic substituent (hac*) was essential for achieving high stereoselectivity, which was attributed to enhanced catalyst‐substrate steric interactions.

**Scheme 15:**
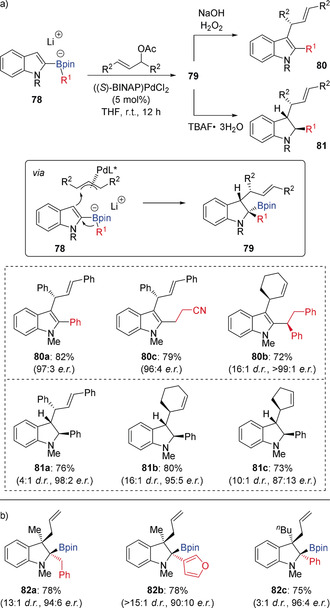
Palladium‐catalyzed enantioselective three‐component coupling.

Electrophilic palladium complexes have also been used to trigger a 1,2‐migration in indole‐derived boronate complexes. Following Ishikura's studies on palladium‐catalyzed allylation of 2‐indolyboronates derived from trialkylboranes,^37^ Ready showed that these reactions could be extended to boronic esters and rendered asymmetric using Pd(BINAP) catalysts (Scheme 15).^38^ The indole‐derived boronate complexes **78** reacted with Pd(π‐allyl) complexes, to form indolin‐2‐yl boronic esters **79** with high levels of diastereo‐, regio‐, and enantioselectivity. The boronic esters products were oxidized with basic hydrogen peroxide to provide the corresponding indoles **80**. Alternately, protodeborylation of benzylic boronic ester products (**79**, R^1^=aryl) with TBAF trihydrate gave 2,3‐disubsituted indolines **81**. Various aryl and alkyl migrating groups could be employed in this asymmetric three‐component coupling, which provided indoline products with three contiguous stereogenic centers. The scope of the reaction was subsequently extended to 3‐alkyl‐substituted indoles by using a Pd/phosphoramidite catalyst system, which enabled the enantioselective formation of indolin‐2‐yl boronic esters **82** with adjacent quaternary stereocenters.^39^

**Scheme 16:**
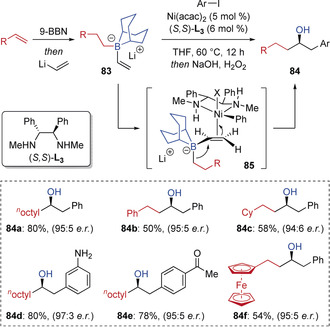
Ni‐catalyzed enantioselective conjunctive cross‐coupling reactions.

Morken has also demonstrated that nickel(II) complexes interact with alkenyl boronates in a similar manner to palladium(II) complexes.^40^ When investigating a one‐pot 9‐BBN hydroboration/enantioselective conjunctive cross‐coupling reaction between alkenes and aryl iodides, they found that the Pd/Mandyphos catalyst system that was optimal for pinacol boronate substrates only provided racemic products when applied to the 9‐BBN‐derived boronates **83** (Scheme 16). However, a nickel catalyst in combination with the diamine ligand (*S*,*S*)‐**L_3_** gave the products **84** in high enantioselectivity. Detailed mechanistic studies indicated that the reaction involves initial oxidative addition of the aryl iodide to Ni^0^ to give a Ni^II^ species, which binds the alkene (forming **85**) to induce 1,2‐migration with stereospecific *anti* addition of the migrating group and Ni^II^ across the alkene. Morken subsequently extended the scope of these nickel‐catalyzed conjunctive cross‐couplings to other electrophiles, including alkyl halides and acid chlorides.^41^

## 3 Stereospecific sp^2^–sp^3^ Coupling of Chiral Boronic Esters with Aromatic Compounds

**Scheme 17:**
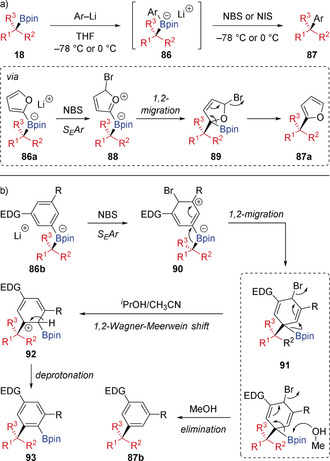
Coupling boronic esters with electron‐rich aromatic compounds.

In 2014, Aggarwal disclosed an efficient and general method for stereospecific sp^2^–sp^3^ couplings of electron‐rich (hetero)aromatics with chiral secondary and tertiary boronic esters (Scheme 17 a).^42^ The reaction occurs by initial reaction of an aryllithium with boronic ester **18** to form aryl boronate complex **86**, followed by treatment with an electrophilic halogenating agent to provide the arylated product **87** in high yield and with complete stereospecificity. This process could be used to introduce various electron‐rich aromatic groups, including 5‐membered ring heteroaromatics and 6‐membered ring aromatics with *meta*‐electron‐donating groups, and was applicable to a broad range of secondary and tertiary boronic esters with different steric demands. In most cases, NBS was the optimal electrophile, with NIS being employed in cases where further halogenation of the electron‐rich aromatic ring occurred. Mechanistically, the addition of NBS to the aromatic ring of boronate complex **86** generates cation **88**. This triggers a stereospecific 1,2‐migration, forming *δ*‐halo allylic boronic ester intermediate **89**, and subsequent elimination/rearomatization leads to the arylated product **87**. Subsequent DFT calculations on the reaction between furyl boronate complex **86 a** and NBS provided evidence for simultaneous electrophilic bromination and 1,2‐migration steps, without formation of the postulated cationic intermediate **88**.^43^

In later studies, it was found that the coupling of 6‐membered ring aromatics was dramatically affected by solvent choice (Scheme 17 b).^43^ Solvent exchange from THF to MeOH led to improved yields of coupled products **87**, which was due to a reduction of the amount of undesired S_E_2 bromination of the C−B bond of **86**. Interestingly, switching to less nucleophilic alcohol solvents promoted an alternative arylation pathway to provide Bpin‐incorporated coupling products **93** with complete stereospecificity. Using an ^*i*^PrOH‐MeCN mixed solvent system resulted in an inefficient nucleophile‐promoted Bpin elimination of dearomatized intermediate **91**, therefore **91** underwent a 1,2‐Wagner–Meerwein shift^44^ of the Bpin moiety to form carbocation **92**, which relieved steric encumbrance and allowed subsequent rearomatization by deprotonation to afford **93**.

**Scheme 18:**
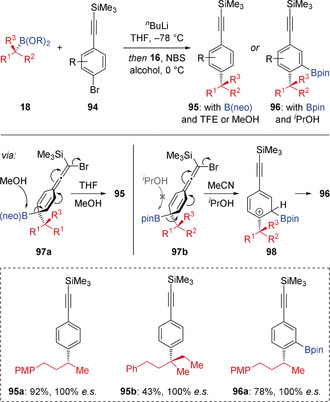
Coupling boronic esters with phenylacetylenes via alkyne activation.

Aggarwal has since expanded this concept of electrophilic‐induced arylation of boronic esters to allow coupling of a range of substituted aromatic rings. For example, phenylacetylene products **95** and **96** could be accessed by coupling between *p*‐lithiated phenylacetylenes (generated by halogen‐lithium exchange of the corresponding bromide **94**) and a range of chiral boronic esters **18** (Scheme 18).^45^ Treatment of the intermediate TMS‐phenylacetylene‐derived boronate complex with NBS results in bromination of the alkyne motif, which triggered a stereospecific 1,2‐migration leading to dearomatized bromoallene intermediate **97**. Using unhindered neopentyl glycol boronic esters and MeOH as solvent, subsequent nucleophile‐promoted elimination and rearomatization of **97 a** occurred, resulting in the formation of coupled product **95**. In contrast, the use of the more hindered pinacol boronic esters and ^*i*^PrOH as the solvent prevented nucleophile‐promoted elimination, therefore 1,2‐Wagner–Meerwein shift of the Bpin moiety occurred instead. This led to carbocation **98**, which, after loss of a proton, furnished the *ortho* Bpin‐incorporated product **96**.

**Scheme 19:**
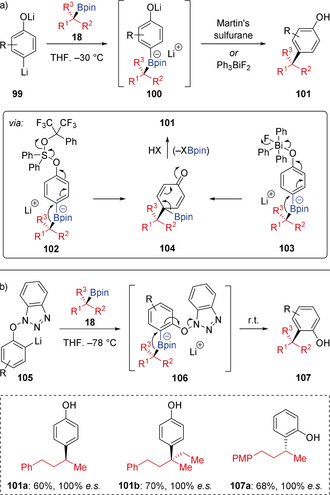
Coupling boronic esters with *ortho*‐ and *para*‐phenols.

*Ortho*‐ and *para*‐substituted phenols provide a different opportunity for triggering 1,2‐migration of aryl boronate complexes (Scheme 19).^46^ In the coupling of *para*‐lithiated phenolates **99** with boronic esters, following formation of boronate complex **100**, 1,2‐migration occurred upon the activation of the phenolate with Martin's sulfurane (Ph_2_S[OC(CF_3_)_2_Ph]) or triphenylbismuth difluoride (Ph_3_BiF_2_), forming boronate complexes **102** and **103**, respectively (Scheme 19 a). Elimination of Bpin from cyclohexandienone **104** then provides the coupled products **101**. This method was less effective for *ortho*‐substituted phenols due to increased steric hindrance, which prevented effective phenolate activation. Interestingly, this limitation was overcome by performing the coupling with lithiated *N*‐phenoxy benzotriazole **105**, where the pre‐incorporated benzotriazole acts as a leaving group (see intermediate **106**) so circumvents the challenging *ortho*‐phenolate activation (Scheme 19 b). 1,2‐Migration successfully occurred at ambient temperature to allow access to *ortho*‐substituted phenol products **107** with complete stereospecificity.

**Scheme 20:**
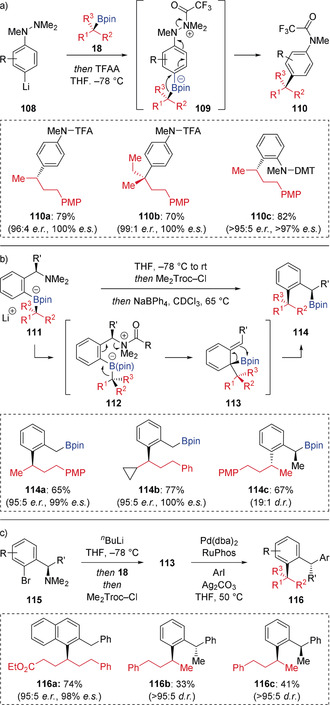
Coupling boronic esters with lithiated arylhydrazines and *ortho*‐lithiated benzylamines. TFA=trifluoroacetyl. DMT=2,2,2‐trichloro‐1,1‐dimethylethoxycarbonyl.

A similar strategy was used by Aggarwal to access aniline products **110** through *N*‐acylation of boronate complexes generated from lithiated *para*‐ and *ortho*‐phenyl hydrazines **108** (Scheme 20 a).^47^ Acylation of the *para*‐hydrazinyl boronate complex with trifluoroacetic anhydride (TFAA) formed acyl ammonium **109**, with subsequent concurrent 1,2‐migration and N−N bond cleavage. After Bpin elimation/rearomatization and further reaction of the resulting amino group with TFAA, the trifluoroacetamide products **110** were isolated in good yield and with complete stereospecificity. For the corresponding *ortho*‐hydrazinyl boronate complexes, changing the *N*‐activator from TFAA to the less reactive 2,2,2‐trichloro‐1,1‐dimethylethyl chloroformate (Me_2_Troc‐Cl) was required to obtain the *ortho*‐aniline products in good yield.

By taking advantage of this *N*‐acylation approach, Aggarwal showed that boronate complexes **111** derived from *ortho*‐benzylamines can also undergo electrophile induced 1,2‐migration (Scheme 20 b).^48^ Treatment of boronate complex **111** with Me_2_Troc‐Cl generated *N*‐acylated intermediate **112**, which triggers a 1,2‐migration/*anti*‐S_N_2′ reaction to form dearomatized intermediate **113**. This step is surprisingly fast and complete within 5 minutes at −78 °C. A subsequent suprafacial Lewis acid mediated 1,3‐borotropic shift of **113** gave enantioenriched *ortho*‐substituted benzylic boronic esters **114** in high yields and stereospecificities. Furthermore, through the use of enantioenriched secondary benzylic amine substrates, it was shown that the *anti*‐S_N_2′ and 1,3‐borotropic shift processes also proceeded with high stereospecificity, which allowed doubly stereospecific reactions to occur when enantioenriched boronic esters were also employed (see product **114 c**). Further work highlighted the synthetic utility of the intermediate enantioenriched dearomatized tertiary boronic esters **113**, which were utilized in rearomatizing allylic Suzuki–Miyaura cross‐coupling reactions to provide complex enantioenriched 1,1‐diarylmethane products **116** with three readily addressable points of diversification (Scheme 20 c).^49^

**Scheme 21:**
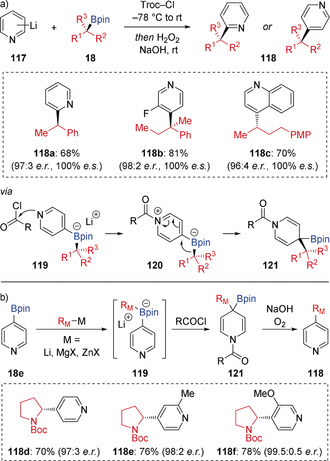
Coupling boronic esters with electron‐deficient N‐heteroaromatics.

In an alternative *N*‐acylation‐induced 1,2‐migration of aryl boronate complexes, Aggarwal developed a general protocol for the stereospecific coupling of chiral secondary and tertiary boronic esters with electron‐deficient N‐heteroaromatics (Scheme 21 a).^50^ After formation of chiral boronate complexes **119** from lithiated 6‐membered ring N‐heterocycles **117** (including pyridines, quinolines and isoquinolines), 1,2‐migration was triggered by N‐acylation with 2,2,2‐trichloroethyl chloroformate (Troc‐Cl), leading to dearomatized tertiary boronic ester **121** via the intermediate *N*‐acyl pyridinium **120**. A one‐pot oxidation/hydrolysis/elimination sequence finally furnished the coupled heteroaromatic products **118** with complete stereospecificity. A modified approach was reported by Ready, in which the pyridyl boronate complexes **119** were generated by adding organometallic reagents to 4‐pyridyl boronic ester **18 e** (Scheme 21 b).^51^ It was shown that, in addition to organolithium reagents, organozinc and Grignard reagents could also be employed in this heteroarylation reaction.

## 4 Electrophile‐Induced 1,2‐Migration of Strained Boronates

**Scheme 22:**
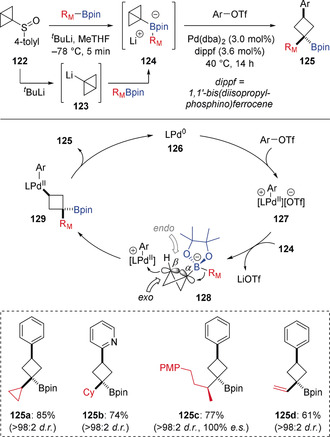
Pd‐catalyzed strain‐release‐driven diastereoselective distal cross‐coupling reaction. R_M_=Migrating group.

It is shown above that electrophilic metal complexes, including Pd^II^ and Ni^II^, can coordinate with the π‐bonds of alkenyl boronate complexes to trigger 1,2‐migration and achieve carbometallation of alkenes (Scheme 11). Although such metal species readily react with C−C π‐bonds, they generally do not react with C−C σ‐bonds. However, Aggarwal has recently reported that cationic palladium(II) complexes can activate σ‐bonds of highly strained boronate complexes to promote 1,2‐migration and achieve σ‐bond carbopalladations (Scheme 22).^52^ To achieve such a process, bicyclo[1.1.0]butyl boronate complexes **124** were prepared from sulfoxide **122** by sulfoxide‐lithium exchange and in situ borylation of the resulting bicyclo[1.1.0]butyllithium (**123**). The high ring strain of the bicyclo[1.1.0]butane (≈66 kcal mol^−1^) weakens the central σ‐bond, and the release of this strain provides significant driving force to allow efficient reaction of **124** with a Pd^II^ catalyst. This enabled a distal cross‐coupling of boronic esters and aryl triflates to provide 1,1,3‐trisubstituted cyclobutanes **125** in high yields and with complete stereospecificity and diastereocontrol. The proposed mechanism involves initial oxidative addition of the aryl triflate to the Pd^0^ catalyst **126** to form the cationic Pd^II^ complex **127**. Reaction of **127** with boronate complex **124** occurs at the more nucleophilic β‐carbon to provide the cyclobutyl palladium intermediate **128**. As 1,2‐migration requires an anti‐periplanar alignment of the migrating group (R_M_) and the breaking C−C bond, this makes the *endo* face of the reactive conformer more sterically hindered, thus the bulky metal complex approaches from the more exposed *exo* face. This forms intermediate **129** with complete diastereocontrol for *syn*‐carbopalladation, which, after stereospecific reductive elimination provides **125** in excellent diastereoselectivity. This interesting strain release‐driven 1,2‐migation of bicyclo[1.1.0]butyl boronate complexes opens up new directions for stereospecific transformations involving 1,2‐migration to sp^3^‐hybridized carbons.

## 5 Summary and Outlook

Organoboron compounds are indispensable in synthetic chemistry, providing a powerful platform for myriad transformations. The stereospecific 1,2‐migration of boronate complexes is one of the most important processes in this area. This can be triggered by a suitable α‐leaving group, oxidation of α‐boryl radicals, or electrophilic activation. As described above, electrophilic activation of boronate complexes can take many different forms and provide access to a diverse array of products from readily available chiral boronic ester. In the case of the Zweifel olefination, reaction of alkenyl boronate complexes with iodine transforms boronic esters into alkenes with high selectivity for inversion of alkene geometry, providing a valuable methodology that has been exploited extensively in total synthesis. This concept has more recently been extended chalcogenation of alkenyl boronate complexes, including selenation, which provides a unique opportunity to switch the stereoselectivity of the Zweifel olefination from inversion to retention. Furthermore, the principles behind the Zweifel olefination have inspired the development of a broad range of arylation and heteroarylation reactions. An important advance in alkenyl boronate complex reactivity has been the development of enantioselective metal‐catalyzed conjunctive cross‐couplings, which have greatly expanded the range of electrophiles that can be employed in electrophile‐induced 1,2‐migration chemistry. This new area in boron chemistry and has since been extended to boronate complexes containing highly strained σ‐bonds in place of π‐bonds, providing further unique opportunities for reaction development.

Future developments could see the application of boronic esters in stereospecific 1,2‐migrations of alkynyl boronate complexes, which have so far been unsuccessful due to their instability. In addition, the development of new electrophilic triggers for various boronate complexes will extend the scope of the chemistry, leading to new opportunities in asymmetric synthesis. While the field of electrophile‐induced 1,2‐migration of boronate complexes is over 50 years old, it remains an exciting area that is continually expanding. It is remarkable that the seminal olefination work by Zweifel in 1967 has inspired so many new methodologies with broad‐ranging applications in synthetic chemistry.

## Conflict of interest

The authors declare no conflict of interest.

## Biographical Information

*Hui Wang was born in Anhui*, *China. He received his B.Sc. (2011) and M.Sc. (2014) degrees in organic chemistry from Zhengzhou University under the supervision of Prof. Xiuling Cui and Prof. Yangjie Wu. After that he joined the group of Prof. Lutz Ackermann, Georg‐August‐University Göttingen (Germany) and finished his PhD in 2019. In the same year, he moved to the University of Bristol (UK) as a postdoctoral fellow to work with Prof. Varinder K. Aggarwal on asymmetric synthesis based on boronate complexes*.

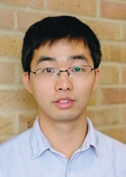

## Biographical Information

*Changcheng Jing obtained his B.A. from the College of Chemistry, Chemical Engineering and Materials Science at Shandong Normal University in 2009. He then conducted his Ph.D. studies under the supervision of Prof. Wenhao Hu in the School of Chemistry and Molecular Engineering of East China Normal University and Prof. Michael P. In 2017 he moved to the group of Prof. Varinder K. Aggarwal at the University of Bristol as a postdoctoral research associate, where his current research interests focus on total synthesis of natural products*.

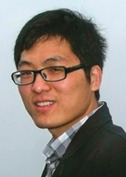

## Biographical Information

*Adam Noble graduated from the University of Nottingham with an M.Sci. degree in Chemistry in 2008. He subsequently gained his Ph.D. (2012) from University College London, working with Prof. Jim Anderson. He then carried out postdoctoral research with Prof. Davis W. C. MacMillan at Princeton University (2012–2014) and with Prof. Varinder K. Aggarwal at the University of Bristol (2014–2017). In 2017, he started his current position as Research Officer in the Aggarwal group at the University of Bristol*.

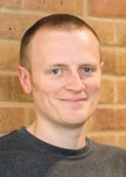

## Biographical Information

*Varinder K. Aggarwal studied chemistry at Cambridge University and received his Ph.D. in 1986 under the guidance of Dr. Stuart Warren. After postdoctoral studies (1986–1988) under Prof. Gilbert Stork, Columbia University, he returned to the UK as a Lecturer at University of Bath. In 1991 he moved to University of Sheffield, where he was promoted to Professor in 1997. In 2000 he moved to the University of Bristol where he holds the Chair in Synthetic Chemistry. He was elected Fellow of the Royal Society in 2012*.